# Metabolic Profiling Study of Yang Deficiency Syndrome in Hepatocellular Carcinoma by H1 NMR and Pattern Recognition

**DOI:** 10.1155/2012/843048

**Published:** 2012-09-27

**Authors:** Xueqiang Huang, Qunwei Chen, Genjin Yang, Weixing Dai, Qingbo Lang, Juan Du, Shikai Yan, Weidong Zhang, Changquan Ling

**Affiliations:** ^1^Department of TCM, Changhai Hospital, Second Military Medical University, Shanghai 200433, China; ^2^Department of Oncology, Zhejiang Provincial Hospital of TCM, Zhejiang 310006, China; ^3^School of Pharmacy, Second Military Medical University, Shanghai 200433, China

## Abstract

This study proposes a ^1^H NMR-based metabonomic approach to explore the biochemical characteristics of Yang deficiency syndrome in hepatocellular carcinoma (HCC) based on serum metabolic profiling. Serum samples from 21 cases of Yang deficiency syndrome HCC patients (YDS-HCC) and 21 cases of non-Yang deficiency syndrome HCC patients (NYDS-HCC) were analyzed using ^1^H NMR spectroscopy and partial least squares discriminant analysis (PLS-DA) was applied to visualize the variation patterns in metabolic profiling of sera from different groups. The differential metabolites were identified and the biochemical characteristics were analyzed. We found that the intensities of six metabolites (LDL/VLDL, isoleucine, lactate, lipids, choline, and glucose/sugars) in serum of Yang deficiency syndrome patients were lower than those of non-Yang deficiency syndrome patients. It implies that multiple metabolisms, mainly including lipid, amino acid, and energy metabolisms, are unbalanced or weakened in Yang deficiency syndrome patients with HCC. The decreased intensities of metabolites including LDL/VLDL, isoleucine, lactate, lipids, choline, and glucose/sugars in serum may be the distinctive metabolic variations of Yang deficiency syndrome patients with HCC. And these metabolites may be potential biomarkers for diagnosis of Yang deficiency syndrome in HCC.

## 1. Introduction 

Hepatocellular carcinoma (HCC) is a serious health problem worldwide. Chinese medicinal herbs have been widely used for treatment of HCC in China and some clinical trials have been done to investigate their effects [1, 2]. All these treatments were based on traditional Chinese medicine (TCM) diagnostics, and syndrome differentiation was the key. As known that TCM diagnostics is often based on observation of human symptoms rather than “microlevel” laboratory tests, therefore, TCM diagnostics often lacks standard and objective diagnostic methods for HCC in TCM practice. At present, many works have been done on diagnostic standards for TCM syndromes of HCC [3]. However, to our knowledge, no work has been done to explore the internal characteristics of TCM syndromes in HCC with a holistic view which is the basic nature of TCM theory. 

Metabonomics, one of modern “-omics” approaches, provides a platform of systems biology to explore the metabolic pathway of biosystem by measuring the metabolic responses to pathophysiological stimuli or genetic modification [4]. It involves the global analysis of the entire metabolic profiling to elucidate the global functional status of the organism, which agrees well with the holistic view of TCM. In TCM theory, TCM syndromes are also the comprehensive responses of a certain stage in the disease process. Therefore, it is reasonable to believe that TCM theory is intrinsically correlated with metabonomics, so using metabonomics technologies is feasible for exploring the biochemical characteristics of TCM syndromes [5].

In our previous works, we established diagnostic principles for TCM basic syndromes of HCC [6, 7]. The patients with HCC are accordingly classified into eight types of basic syndromes, that is, Qi stasis, damp stasis, blood stasis, heat, Qi deficiency, blood deficiency, Yin deficiency, and Yang deficiency. As one of these basic syndromes, Yang deficiency syndrome is a special syndrome which has typical clinical manifestations. The symptoms of Yang deficiency syndrome, mainly including intolerance of cold and cool limbs, are easy to be distinguished even for a junior physician, and the diagnosis of Yang deficiency syndrome is hardly controversial in TCM diagnostics. Therefore, in this study, we chose Yang deficiency syndrome patients with HCC as research subjects and tried to investigate the serum metabolic variations and discover the biochemical characteristics of Yang deficiency syndrome in HCC using a proton nuclear-magnetic-resonance- (^1^HNMR-) based metabonomic approach, so as to provide evidences for objectification and standardization in diagnosis of TCM syndromes in HCC.

## 2. Materials and Methods

### 2.1. Patient Cohort and Classification

All experimental procedures were approved by the ethics and research committee of Changhai Hospital, Second Military Medical University, China. After obtaining informed consent, we collected a total of 42 serum samples from previously untreated HCC patients included in this study (see Table 1). Clinical diagnosis of the patients was performed using standard imaging and pathology criteria. Basic syndrome types of 42 patients with HCC were classified by TCM syndrome qualitative diagnosis criteria and quantitative diagnosis model for HCC patients into two groups, including Yang deficiency syndrome (YDS-HCC) group (21 cases) and non-Yang deficiency syndrome (NYDS-HCC) group (21 cases). Between two groups, the differences of gender, age, complication, and clinical stage were not significant (*P* > 0.05) (see Table 1).

### 2.2. Sample Preparation

To avoid the introduction of any analytical bias due to sample preparation, all samples were collected from the patients in the Department of TCM of Changhai Hospital and were processed consistently in the following manner. Venous blood samples were collected in the morning preprandially, after overnight fasting, using blood collection vacuum tubes with silicone coated interior and no additive (products of BD Company, USA, catalog no. 366431). About 2.0 mL blood was collected and then left clotted for 2 h at room temperature. After this, samples were centrifuged at 3500 rpm for 15 min. Serum was collected and immediately stored at −80°C until being used for metabonomic analysis. Preparation of serum samples for metabonomic analysis by ^1^H-NMR was performed manually according to Beckonert et al. [8]. After being defrosted at room temperature, four hundred microliters of each sample were mixed with 50 mL D_2_O for locking signal and with 50 mL phosphate buffer solution (0.2 M Na_2_HPO_4_/0.2 M NaH_2_PO_4_; pH 7.4) to minimize pH variations of the serum samples. Then, TPS (3-trimethylsilylpropanesulfonate sodium; 0.048% final concentration) was added as an internal standard. Finally, the samples were added to a 5 mm NMR tube and NMR acquisition was performed immediately.

### 2.3. NMR Acquisition

The NMR spectra were acquired using a Bruker Avance II 600 spectrometer (Bruker Biospin, Rheinstetten, Germany). The ^1^H-NMR spectra of serum samples were collected at a constant temperature of 300 K (27°C) using the relaxation edited Carr-Purcell-Meiboom-Gill (CPMG) pulse sequence to facilitate the detection of low molecular weight species and using a solvent presaturation pulse sequence to suppress the residual water resonance. Free induction decays (FIDs) were collected into 64 k data points, with a spectral width of 7289 Hz and an acquisition time of 2.04 s, giving a total pulse recycle delay of 3.04 s. The FID values were multiplied by an exponential weighting function equivalent to a line broadening of 0.3 Hz prior to Fourier transformation. 

### 2.4. Data Processing and Statistical Analysis

All spectra were baseline corrected using multipoint baseline correction method, and chemical shifts were adjusted with reference to TPS. The chemical shift region *δ*4.60–5.06 was removed to eliminate any spurious effects of variability on the suppression of the water resonance. Peak detection and peak matching were performed using programs coded by Matlab 7.0 (The Mathworks Inc., Natick, MA, USA), and a data matrix containing all peak intensities was thus generated. The data were logarithm transformed and centered for partial least squares discriminant analysis (PLS-DA) using Matlab 7.0. In PLS-DA, the pathologic differences in patients can be visualized in score plot and metabolites responsible for the differences could be statistically identified in loading plot using Hotelling's *T* ^2^ test [9]. All results presented are described as mean ± SD. Statistical significance of the data was determined using the non parametric Mann-Whitney *U* test or *t* test, where appropriate, *P* values < 0.05 were considered to be significant. Metabolite assignments were performed using NMR metabolic profiling databases (http://www.ebi.ac.uk/nmrshiftdb or http://nmrshiftdb.org/), and if needed, with reference to any available assignments in the literature [10–12].

## 3. Results

**Figure 1:**
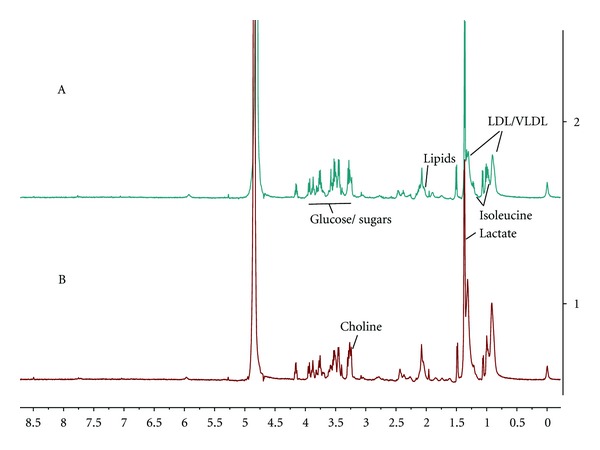
Representative ^1^H NMR spectra of serum from YDS-HCC patients (A) and NYDS-HCC patients (B); LDL, low-density lipid; VLDL, very low-density lipid.

**Figure 2:**
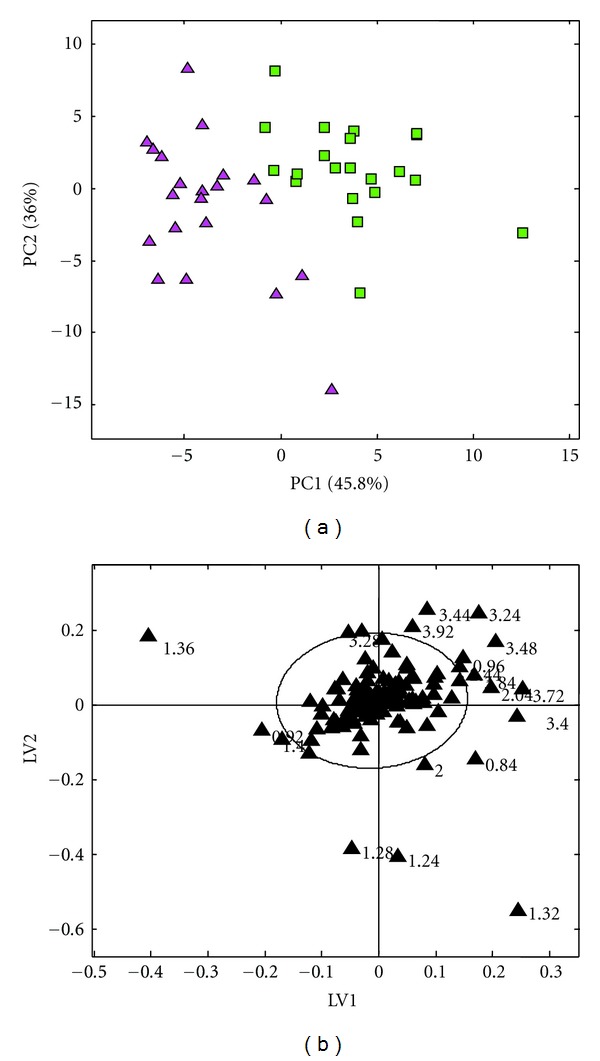
Results after PLS-DA using the ^1^H NMR spectra of serum from HCC patients. (a) PLS-DA score plot shows a clear separation between Yang deficiency syndrome patients with HCC (YDS-HCC) (■) and non-Yang deficiency syndrome patients with HCC (NYDS-HCC) (▲). (b) Examination of the corresponding loading plot indicated that those variances out of the ellipse were responsible for the separation of YDS-HCC and NYDS-HCC patients marked with chemical shift (ppm).

Typical ^1^H NMR spectra of the serum samples were shown in Figure 1. By data preprocessing, a total of 221 peaks were resolved for each serum sample. To determine whether any Yang deficiency syndrome-related variability existed within the data, PLS-DA was used. As shown in PLS-DA scores plot (Figure 2(a)), samples from different groups were well separated along the first PLS component, which indicates that NMR-based metabolic profiling could reveal characteristic pathological alterations in serum from YDS-HCC and NYDS-HCC patients.

As shown in PLS-DA loading plot (Figure 2(b)), eighteen important resonances responsible for the separation were selected. It displayed not only the differentiation between the two groups but also the specific compounds resulting in the separation. These resonances were structurally identified as low-density lipid/very low-density lipid (LDL/VLDL) (0.84 ppm, 0.92 ppm, 1.24 ppm, 1.28 ppm, 1.32 ppm), isoleucine (0.96 ppm), lactate (1.36 ppm, 1.40 ppm), lipids (2.00 ppm, 2.04 ppm), choline (3.24 ppm), and glucose/sugars (3.28 ppm, 3.40 ppm, 3.44 ppm, 3.48 ppm, 3.72 ppm, 3.84 ppm, 3.92 ppm). As shown in Table 2, aftere being confirmed by Mann-Whitney *U* test or *t* test, intensities of these six types of metabolites were changed markedly between different groups (*P* < 0.05 or *P* < 0.01). Differences in metabolite intensities mirrored those observed in the PLS-DA models with all the differential metabolite observed, including LDL/VLDL, isoleucine, lactate, lipids, choline, and glucose/sugars, being lower in YDS-HCC patients compared to NYDS-HCC patients.

## 4. Discussion

The serum metabolite profiling represents a collective “snapshot” of metabolic alterations in the biochemical pathways of the entire body induced by a plethora of factors, including disease, life habits (e.g., diet, smoking, and exercise), gender, drug intake, and environmental factors [10]. In TCM theory, syndrome also is a comprehensive reflection of physical status in one stage of disease. Different patients with the same disease might have different clinical signs and symptoms as well as TCM tongue condition and pulse condition. Yang deficiency syndrome is a special syndrome with typical clinical manifestations, mainly including intolerance of cold, cool limbs, pale tongue, and powerless pulse. To reveal the internal characteristics behind these special clinical manifestations in HCC patients is not only significant for objective diagnosis of TCM syndromes but also significant for understanding the heterogeneity of HCC patients.

Efforts to understand the biochemical characteristics of Yang deficiency syndrome in HCC or other diseases through analyzing various kinds of biological indicators have been made by a number of groups [13, 14]. However, to the best of our knowledge, this paper presents the first examination of serum metabolites in the context of Yang deficiency syndrome in HCC with a global view. We have examined the potential differentiating capacity of an ^1^H-NMR-based metabonomic approach for HCC patients as classified by Yang deficiency status. 

Supervised multivariate analysis in the form of PLS-DA enabled good separation of YDS-HCC and NYDS-HCC patients (see Figure 2(a)). PLS-DA statistical analysis indicated that the metabolic profiling of one particular YDS-HCC patient was more similar to that of YDS-HCC patients. And several Yang deficiency syndrome-related variations determined the distinction between YDS-HCC and NYDS-HCC (see Figure 2(b)). Examination of corresponding loading plots showed that the main metabolic variations in the data set were derived from various metabolic signals. The metabolites responsible for the separation of the YDS-HCC from the NYDS-HCC patients were structurally identified as LDL/VLDL, isoleucine, lactate, lipids, choline, and glucose/sugars. And the intensities of these six types of metabolites were lower markedly in YDS-HCC patients compared to NYDS-HCC patients (see Table 2). These six types of metabolites get involved in multiple metabolisms, including amino acid metabolism, lipid metabolism, glycometabolism, and energy metabolism. The results implied multiple Yang deficiency related biochemical pathways lose balance in HCC patients. 

In ^1^H-NMR serum profiling, lower intensities of LDL/VLDL, lipids, and choline in YDS-HCC than NYDS-HCC patients are correlated to lipid metabolism. LDL and VLDL play an important role in lipid metabolism as a transporter for cholesterol and triglyceride, respectively [15]. Lipids, the general term of fats and lipoids, are the best storage form of redundant energy and play the wide biological functions as different structural styles. Choline can increase fatty acid utilization and prevent abnormal accumulation of fat in liver. Choline is involved in the synthesis of creatine and indirectly involved in muscle energy metabolism [16]. Usually, lipid metabolism is abnormal in HCC due to the deficient abilities of both lipid catabolism and anabolism [17]. Lower intensities of LDL/VLDL, lipids, and choline in YDS-HCC patients implied the lower level of lipid metabolism in YDS-HCC patients. In TCM theory, Yang deficiency syndrome patients usually have a poor appetite and failed to utilize the food essence normally. Lipids and lipoproteins are composed by lots of materials from food. It is consistent with the results of lower level lipids and lipoproteins in Yang deficiency patients in our study. Yang deficiency can reduce the ability of transporting food essence to all the body. Lower intensities of lipoproteins, like LDL and VLDL, reflect the deficiency of transport capacity for nutrients. Intolerance of cold and cool limbs, the typical clinical manifestations of patients with Yang deficiency syndrome, is the outward appearance of deficient energy metabolism and fat reserves. Lower levels of lipids, lipoproteins, and choline reflect the decrease of ability to use and reserve the lipid energy substances. It is also coincident with the results of lower level of lipids, lipoproteins, and choline in Yang deficiency syndrome patients in our study. However, because of the poor analysis capabilities for lipids metabolites by the means of CPMG pulse sequence in ^1^H-NMR spectra, it is necessary to analyze the relations between lipids metabolites and Yang deficiency syndrome by longitudinal eddy-delay (LED) pulse sequence in our further study. 

In our examination, the differential metabolite involved in amino acid metabolism is isoleucine. Isoleucine, one of branched-chain amino acids, can synthesize protein for muscles and can transform into glucose for providing energy in the status of starvation or strenuous exercise. Branched-chain amino acids usually decrease in severe liver diseases. Supplement branched-chain amino acids can modify negative nitrogen balance and cachexia in HCC patients [18]. There is few research to explore the relation between Yang deficiency syndrome and amino acid metabolism. However, one research found that the serum concentration of branched-chain amino acids in the elderly was lower than the youth under the same nutritional intake. And the reduction of branched-chain amino acids was accompanied with decreasing immune status and lower level of hormone [19]. In TCM theory, Yang deficiency syndrome is similar to natural aging. In other words, Yang deficiency syndrome usually means premature senility. Therefore, the level of branched-chain amino acids might partly reflect the pathological feature of Yang deficiency syndrome. And low level of isoleucine might be one of potential biochemical characteristics for Yang deficiency syndrome patients with HCC. 

Glucose is the main source of energy for the human body and in a high-consuming status in tumors [20]. Lactate is an important intermediate of anaerobic glycolysis in glucose metabolism. Lactate can send back to liver or muscle and convert into glcogen. In normal condition, anaerobic glycolysis is intensive in organs or cells with high energy-consuming or lacking of mitochondria, such as adrenal medulla, neurons, and blood red cells and can keep a stable lactate concentration in blood [21]. Anaerobic glycolysis is also an important way of energy supply for tumor cells. Lactate is not only a metabolite of energy metabolism but also a kind of energy source for tumor cells [22]. In our examination, the intensities of both glucose/sugar and lactate decreased in Yang deficiency syndrome patients with HCC. It might mean that the energy metabolism of the organs or cells lacking of mitochondria or with high energy-consuming were weakened, but which one plays the leading role was not clear. On the other hand, glucose/sugar, lactate, lipids, and amino acids are all the energy source, and these reduced metabolites might imply high consume of energy in Yang deficiency syndrome patients with HCC.

In summary, this study provided an ^1^H NMR-based metabonomic approach for exploring the biochemical characteristics of Yang deficiency syndrome based on the integral information about serum metabolites. Yang deficiency syndrome in HCC is correlated to the unbalance of multiple metabolisms. This metabonomic approach to study Yang deficiency syndrome in HCC may lead to better understand the heterogeneity of HCC. It is also beneficial to understand individualized treatment. The discussion regarding metabolic differences between patient groups in this study is far from exhausted due to few studies on Yang deficiency syndrome by tools of systems biology. We need to accumulate more data of TCM syndrome study by systems biology technology, so as to provide evidence of the objectivity of TCM syndrome. 

## 5. Conclusions

In this work, a metabolic profiling study of Yang deficiency syndrome in HCC was performed using ^1^H NMR and pattern recognition techniques, which has been proven to be an efficient approach to investigate the biochemical characteristics of Yang deficiency syndrome. With the application of PLS-DA, six types of metabolites responsible for the separation based on the status of Yang deficiency syndrome were structurally postulated. These metabolites included LDL/VLDL, isoleucine, lactate, lipids, choline, and glucose/sugars, which were mainly involved in lipid, amino acid, and energy metabolisms. The decreased intensities of these metabolites imply that multiple metabolisms, mainly including lipid, amino acid, and energy metabolisms, are unbalanced or weakened in Yang deficient syndrome patients with HCC. The results indicate that the NMR-based metabonomic approach is a promising technique for understanding the internal characteristics of TCM syndromes, and the potential biomarkers for diagnosis of TCM syndromes. 

## Figures and Tables

**Table 1:** Basic clinical feature of hepatocellular carcinoma patients with different syndrome.

Group	Number (*n*)	Gender (*n*)		Age ($\overset{-}{x}\pm \text{SD}$)	HCC clinical staging (*n*)						Child-Pugh grading (*n*)		
		Male	Female		Ia	Ib	IIa	IIb	IIIa	IIIb	A	B	C
YDS-HCC	21	18	3	55.76 ± 12.79	3	0	1	4	11	2	14	5	2
NYDS-HCC	21	19	2	58.10 ± 7.31	3	1	1	6	9	1	14	6	1

HCC: hepatocellular carcinoma; YDS: Yang deficiency syndrome; NYDS: non-Yang deficiency syndrome.

**Table 2:** Assignment and intensities of metabolites in serum responsible for PLS-DA separation between YDS-HCC and NYDS-HCC patients.

Metabolite	*δ* ^ 1^H (ppm)^a^	Assignment	Multiplicity^b^	Intensity^c^ (10^3^)		*P* value^d^
				YDS-HCC (*n* = 21)	NYDS-HCC (*n* = 21)	
LDL/VLDL	0.84, 0.92, 1.24, 1.28, 1.32	CH_3_(CH_2_)_*n*_/CH_3_CH_2_CH_2_C=	m	67.23 ± 32.64	115.66 ± 47.33	0.000
Isoleucine	0.96	*δ*-CH_3_	t	8.27 ± 3.86	13.57 ± 6.20	0.002
Lactate	1.36, 1.40	CH_3_	d	19.44 ± 15.19	53.44 ± 25.92	0.000
Lipids	2.00, 2.04	CH_2_C=C	m	9.45 ± 3.73	13.89 ± 5.04	0.002
Choline	3.24	N(CH_3_)_3_	s	10.23 ± 5.53	16.05 ± 9.04	0.016
Glucose/sugars	3.28, 3.40, 3.44, 3.48, 3.72, 3.84, 3.92	Various	m	46.05 ± 27.74	68.39 ± 40.92	0.045

HCC: hepatocellular carcinoma; YDS: Yang deficiency syndrome; NYDS: non-Yang deficiency syndrome; PLS-DA: partial least square discriminatory analysis; LDL: low-density lipid; VLDL: very low-density lipid.

^a^Chemical shifts were reported with reference to 3-trimethylsilylpropanesulfonate (TPS) singlet resonance at 0.000 ppm.

^b^Multiplicity: s: singlet; d: doublet; t: triplet; m: multiplet.

^c^Data are expressed as mean ± SD.

^d^ *P* value calculated by Mann-Whitney *U* test or *t* test.
